# Selenium-Biofortified Strawberries Improve Glucose Homeostasis and Hepatic Function: A 30-Day Randomized Controlled Trial in Healthy Adults

**DOI:** 10.3390/nu18132078

**Published:** 2026-06-25

**Authors:** Sonya Vasto, Luigi Di Rosa, Vincenzo Ferrantelli, Antonino Salvatore Fiore, Carola Pia Giordano, Alessia Cannizzaro, Leo Sabatino, Andrea Macaluso, Rosalia Caldarella, Gaetano Felice Caldara, Sara Baldassano

**Affiliations:** 1 Department of Biological, Chemical and Pharmaceutical Sciences and Technologies, University of Palermo, 90133 Palermo, Italy; sonya.vasto@unipa.it (S.V.); luigi.dirosa@unipa.it (L.D.R.); antoninosalvatore.fiore@community.unipa.it (A.S.F.); carolapia.giordano@community.unipa.it (C.P.G.); alessia.cannizzaro03@community.unipa.it (A.C.); 2 Experimental Zooprophylactic Institute of Sicily, Via Gino Marinuzzi 3, 90129 Palermo, Italy; vincenzo.ferrantelli@izssicilia.it (V.F.); andrea.macaluso@izssicilia.it (A.M.); gaetanofelice.caldara@unipa.it (G.F.C.); 3 Dipartimento Scienze Agrarie, Alimentari e Forestali (SAAF), University of Palermo, Viale Delle Scienze, Ed. 5, 90128 Palermo, Italy; leo.sabatino@unipa.it; 4 Department of Laboratory Medicine, “P. Giaccone” University Hospital, 90127 Palermo, Italy; liacaldarella@virgilio.it

**Keywords:** micronutrients, supplementation, biofortification, nutritional intervention

## Abstract

**Background**: Selenium is an essential trace element for humans that plays a key role in glucose homeostasis and hepatic function. Biofortification is a sustainable agricultural technique able to increase micronutrients and reduce pesticides in crops. **Purpose**: The present study aimed to examine whether the consumption of strawberries biofortified with selenium in a healthy population for 30 days would increase the endogenous selenium concentration, and whether and to what extent it would impact glucose homeostasis and hepatic function. **Methods**: Thirty-five healthy participants, male and female, were divided by double-blinding into three different groups that received control strawberries (100 g/day), selenium-biofortified strawberries (100 g/day) or selenium in tablets (100 µg/day) for 30 consecutive days. Blood samples were collected at the beginning (T0, baseline) and at the end of the nutritional intervention (T1), and the groups were compared for differences in serum selenium concentrations, glucose homeostasis aminotransferase (AST), alanine aminotransferase (ALT), gamma-glutamyl transferase (GGT), and albumin (ALB). **Results**: Biofortification increased the selenium concentration in strawberries by 419%. Supplementation with biofortified strawberries increased serum selenium levels by about 73.6%, while standard selenium supplementation showed no statistically significant effect. Selenium-biofortifies strawberries reduced fasting glucose and insulin, and improved insulin sensitivity and β cell function. They also reduced AST and GGT within the physiological range. **Conclusions**: These data suggest that supplementation for 30 days with selenium-biofortified strawberries is safe and is associated with favourable changes in markers of glucose regulation. Selenium supplementation, at the standard market dose of 100 µg/day, demonstrated no significant clinical differences during the studied period.

## 1. Introduction

In recent years, the prevalence and incidence of type 2 diabetes mellitus (T2DM) among children, adolescents and adults under 40 years of age have significantly increased [1]. These trends raise serious public health concerns, as evidence indicates that early-onset T2DM is associated with a more severe disease phenotype. Early onset of T2DM means a rapid decline in β-cell secretory function, an earlier need for insulin therapy and an increased lifetime risk of adverse long-term outcomes [2]. It also increases the risk of metabolic-dysfunction-associated steatotic liver disease (MASLD) [3]. Thus, solutions for prevention of this non-communicable disease, particularly through dietary strategies, are necessary. Dietary approaches are key to prevention, and biofortification represents a promising dietary strategy. Biofortification embodies the concept of food for the future because it enhances the nutrient content of crops, promoting healthier nutrition [4]. Biofortification of plants with minerals consists of providing micronutrients through fertilization to enhance the mineral uptake of the crops. This process not only increases the nutritional value of food but also enhances plant stress tolerance and reduces fertilizer use [5]. The result is higher-quality crops with more resilience and better nutritional value [6]. Deficiencies in essential minerals occur not only in low-income countries but also in developed countries due to monotonous diets, dietary restrictions (e.g., vegan or vegetarian), and a lack of nutritional education [7]. Selenium is a trace element that is essential for normal physiological processes because it plays a crucial role in various aspects of human health. It is a component of selenoproteins and is required for adequate function of glutathione peroxidase as an enzyme cofactor. Selenium deficiency affects an estimated 15% of the world population, increasing the risk of numerous inflammatory disorders primarily affecting glucose and liver function [8]. To support optimal biological functioning, the Recommended Dietary Allowance (RDA) for selenium is 70 µg/day for men and 55 µg/day for women, but this level is considered low, with some studies suggesting a minimum requirement of 90 µg/day for adults. The tolerable upper intake level set by the World Health Organization for selenium in adults that is 400 µg/day [9]. Climate change, together with agricultural practices, is depleting soil selenium levels over time [10]. The present study aimed to examine whether daily intake of selenium-biofortified strawberries over a period of 30 days would increase physiological selenium levels and influence biomarkers of glucose homeostasis and hepatic function in a healthy population. We chose a 30-day intervention period because 30 days is sufficient to detect changes in certain biomarkers, such as endogenous selenium levels, fasting glucose, insulin, and liver enzymes, allowing researchers to assess safety and tolerability for several days, while also making it easier to recruit and retain participants. Thus, thirty-five healthy participants, male and female, were divided by double-blinding into three different groups that received control strawberries (100 g/day), selenium-biofortified strawberries (100 g/day) or selenium in tablets (100 µg/day) for 30 consecutive days. Blood samples were collected at the beginning (T0, baseline) and at the end of the nutritional intervention (T1), and the groups were compared for differences in selenium concentrations, glucose homeostasis and markers of hepatic function.

## 2. Materials and Methods

### 2.1. Strawberry Agronomic Trial

Strawberries (*Fragaria × ananassa* cv. ‘Florida fortuna’) were cultivated within a polyethylene-covered tunnel in the experimental field of the University of Palermo’s Department of Agricultural, Food and Forestry Sciences, near Marsala (Trapani province, Sicily). Plants were transplanted into 25 L grow bags filled with coconut fibre (Agripan cocco, Perlite italiana, Milan, Italy), obtaining a plant density of 8 plants per m^2^. The following nutrient solution was employed: 10 mM of N-NO_3_, 1 mM of N-NH_4_, 1 mM of P-PO_4_, 5.50 mM of K, 3.50 of Ca, 1.2 mM of Mg, 2 mM of S-SO_4_, 20 µM of Fe, 30 µM of B, 1 µM of Cu, 5 µM Zn, 10 µM of Mn and 1 µM of Mo. Standard soilless strawberry cultivation practices were followed throughout the growing cycle. Every 10 days starting from 20 days after transplant, half of the plants were sprayed with tap water, while the other half were biofortified with 4 µmol L^−1^ of selenium, administered in form of sodium selenate, using 0.2 L of solution per square metre. The field experiment was organized as a random complete block design, with three replications per block, each containing 500 plants. After harvesting, fruits from the control and treated plots were immediately transferred to the University of Palermo’s Department of Biological, Chemical and Pharmaceutical Sciences and Technologies for the subsequent clinical trial.

### 2.2. Analysis of Selenium Levels

The selenium levels in the strawberries were determined using inductively coupled plasma mass spectrometry (7700x series ICP-MS, Agilent Technologies, Santa Monica, CA, USA). One-millilitre serum samples underwent digestion in an Ultrawave digestion system (Milestone, Sorisole, Italy), employing 3 mL of 60% (*v*/*v*) ultrapure nitric acid and 5 mL of deionized water within pre-decontaminated polytetrafluoroethylene vessels. Following digestion, the samples were diluted to 50 mL with ultrapure (milliQ) deionized water before ICP-MS analysis. Instrument parameters were set as follows: nebulizer carrier gas flow, 1.2 L/min; plasma gas flow, 15 L/min; reflected power < 5; RF power, 1550 W. Calibration was performed using a certified reference standard from Agilent, USA. Calibration curves, constructed through linear interpolation of at least seven points from seven standard solutions and white calibration, were utilized, with a maximum error of 5% on individual standards and a correlation coefficient (r^2^) > 0.999. Validation parameters, including limit of detection (LOD), limit of quantification (LOQ), repeatability, recovery, and uncertainty, were assessed. LOD and LOQ values, 0.0004 mg kg^−1^ and 0.0013 mg kg^−1^, respectively, were calculated based on ten replicates of serum samples spiked at 0.005 mg kg^−1^ (level 1), 0.010 mg kg^−1^ (level 2), and 0.05 mg kg^−1^ (level 3). The repeatability was 0.001 mg kg^−1^ at level 1, 0.001 mg kg^−1^ at level 2, and 0.04 mg kg^−1^ at level 3. Recoveries of 104.3% were achieved across all levels, with uncertainty values of 0.001 mg kg^−1^ at level 1, 0.001 mg kg^−1^ at level 2, and 0.05 mg kg^−1^ at level 3. The measuring field for selenium concentration was between 0.002 and 1.0 mg kg^−1^.

### 2.3. Study Participants

**Figure 1:**
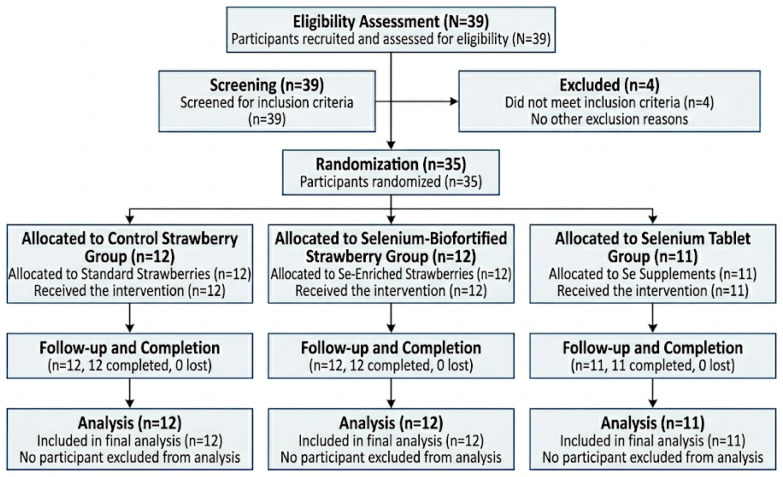
CONSORT-style flow diagram of participant recruitment, screening, and allocation. A total of 39 individuals were assessed for eligibility; 4 were excluded for not meeting the inclusion criteria. Thirty-five participants were randomized into three groups: control strawberries (*n* = 12), selenium-biofortified strawberries (*n* = 12), and selenium tablets (*n* = 11). All participants completed the 30-day intervention and were included in the final analysis.

This study employed a randomized, placebo-controlled design, approved by the Ethics Committee of the University of Palermo Hospital P. Giaccone (protocol number 02/2020) and conducted in accordance with the Declaration of Helsinki. All participants provided written informed consent prior to inclusion, with recruitment commencing in January 2024. The study was registered at clinicaltrials.gov NCT06875674 (12 March 2025). An a priori power calculation, based on previous studies [11,12,13] and utilizing a 5% statistical significance level and 20% probability, suggested a sample size of eight participants per group for fasting insulin level estimations. To mitigate type two error risks and enhance evaluation power for secondary outcomes, a minimum of ten participants per group was targeted. Thirty-nine healthy volunteers (22 females and 17 males) were enrolled. The recruitment and group assignment processes are outlined in Figure 1.

A team of physicians and nutritionists provided individual instruction on completing food and lifestyle diaries, emphasizing the importance of maintaining consistent habits throughout the study period [14].

Participants were instructed to maintain their habitual dietary intake and lifestyle behaviours, including physical activity levels, throughout the entire study period. To ensure consistency and accuracy, participants received detailed guidance on how to complete food diaries.

Dietary intake was recorded for 8 days prior to the intervention and continuously throughout the 30-day study period. These records were used to monitor adherence, compliance, and any potential changes in dietary habits during the intervention. Food diaries were periodically reviewed by trained staff and complemented by dietary re-calls to verify completeness and accuracy. All dietary data were analysed to confirm that no significant differences occurred between groups in total energy intake, macronutrients, or micronutrients, apart from the selenium provided through the experimental interventions [12].

Although strict dietary standardization was not imposed, this approach allowed the study to reflect real-life conditions while minimizing potential dietary confounding.

Nutritionists analysed these dietary intakes using WinFood 3 software to assess energy, macronutrient, micronutrient, water, and fibre consumption, ensuring subjects maintained their usual dietary and lifestyle habits during supplementation to verify homogeneity in the groups as previously reported [15], as shown in Table 1. Furthermore, compliance, adherence and strawberry tolerability were reported successfully. No adverse effects or gastrointestinal problems were recorded.

Eligibility criteria included the absence of gastrointestinal, cardiac, blood, and metabolic disorders, viral infections, medication use (including supplements and hormones), pregnancy, and breastfeeding, as detailed in Table 2.

### 2.4. Design of the Study and Experimental Procedure

**Figure 2:**
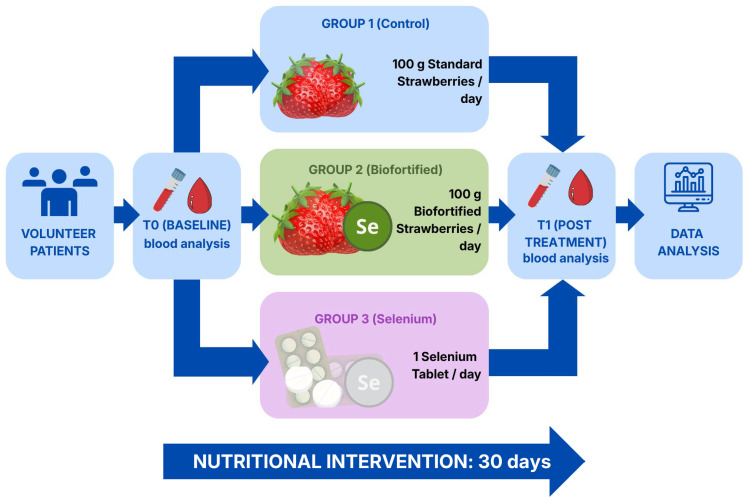
Study design and experimental timeline. Participants were randomly assigned to one of three groups: control strawberries (100 g/day), selenium-biofortified strawberries (100 g/day), or selenium tablets (100 µg/day). The intervention lasted 30 days. Blood samples were collected at baseline (T0) and at the end of the intervention (T1) for biochemical analyses.

Participants attended the ambulatory Nutrition Age and Bone (NABbio) clinic at the University of Palermo’s STEBICEF department between 7:00 and 8:00 a.m., following a 12 h overnight fast. Venous whole blood samples were collected for serum and plasma analysis, and anthropometric measurements, including weight, height, muscle and fat mass, were recorded for everyone. A computer-generated random number program (Excel) was used to randomize participants into control and experimental groups, with a third party assigning coded strawberries to match the random numbers. This double-blind procedure ensured that medical staff, investigators, and participants remained unaware of group allocation throughout data collection, analysis, and sample assessment. Participants in the experimental group received approximately one kilogram of strawberries, with instructions for storage and consumption of 100 g daily for 7 days. They received fresh strawberries each week until the end of the experiments for a total of 30 days of treatment. A total of 39 healthy male and female participants were initially enrolled and randomized. Thirty-five participants completed the 30-day double-blind intervention, divided into three groups: a control group (*n* = 12; 7 females, 5 males) receiving 100 g/day of non-biofortified strawberries; a selenium-biofortified strawberry group (*n* = 12; 7 females, 5 males) receiving 100 g/day of biofortified strawberries; and selenium tablet group (*n* = 11; 6 females, 5 males) receiving 100 µg/day of selenium (Figure 2).

Blood samples were collected at baseline (T0) and at the end of the intervention (T1) to assess serum selenium, glucose, insulin, HOMA-IR, insulin sensitivity, β-cell function, and liver enzymes (AST, ALT, GGT, and ALB). All samples were anonymized with code numbers to protect participant identity during and after data collection. Serum was obtained by collecting blood into tubes without anticoagulant and centrifuging at 1300× *g* for 15 min at room temperature. Glucose, insulin, and hepatic metabolism markers were then measured using an automated Roche COBAS c503 analyser, utilizing commercially available assays from Roche Diagnostics (Indianapolis, IN, USA) as previously described [11,12,13]. Albumin (ALB) was measured by immunoturbidimetric assay. Gamma-glutamyl transferase (GGT) was measured by colorimetric assay. Aspartate aminotransferase (AST) and alanine aminotransferase (ALT) were measured by IFCC (International Federation of Clinical Chemistry and Laboratory Medicine) pyridoxal 5′-phosphate activation assay. Insulin resistance (HOMA-IR), β-cell function (HOMA-% β) and insulin sensitivity (HOMA-%S) were calculated as previously reported [11].

### 2.5. Statistical Analysis

To compare selenium levels between control and treated strawberries, Student’s *t*-test was employed. For the human study, baseline comparisons between the study groups were conducted using Student’s *t*-test, while differences between baseline (T0) and post-intervention (T1) measurements were analysed using one-way ANOVA followed by Tukey’s post hoc test, all performed with GraphPad Prism software 6. Statistical significance was defined as a *p*-value ≤ 0.05. Data are presented as mean ± standard deviation (S.D.). The corresponding effect-size estimates (Cohen’s d) for all outcome measures and confidence intervals are provided in Supplementary Table S1 In addition, to account for the repeated-measures design, a two-way mixed ANOVA (within-subjects factor: time; between-subjects factor: group) was performed for each primary outcome, with partial eta-squared as the effect-size estimate; the corresponding results are reported in the Supplementary Materials (Table S2).

## 3. Results

### 3.1. Selenium Concentration in Biofortified Strawberries

The selenium concentration was measured to verify the efficacy of the biofortification process. The plants from plots treated with 4.0 µmol selenium L^−1^ were used for the nutritional intervention. The amount of selenium in control strawberries was 27 ± 3.3 µg/100 g of fresh weight (fw) compared to 140 ± 7.7 µg/100 g fw in biofortified strawberries, confirming a significant increase (*p* = 0.003) of 418.5%.

### 3.2. Anthropometric Parameters

The study was conducted in a cohort of 35 healthy adults, 20 females and 15 males. Anthropometric characteristics were analysed across all groups. No significant difference was observed in weight, height, body mass index, or percentage of lean and fat mass between the selenium strawberry group, the selenium tablet group and the control group or between baseline and T1 within the groups (Table 3).

### 3.3. Nutritional Intervention with Selenium-Biofortified Strawberries and Serum Concentration of Selenium

**Figure 3:**
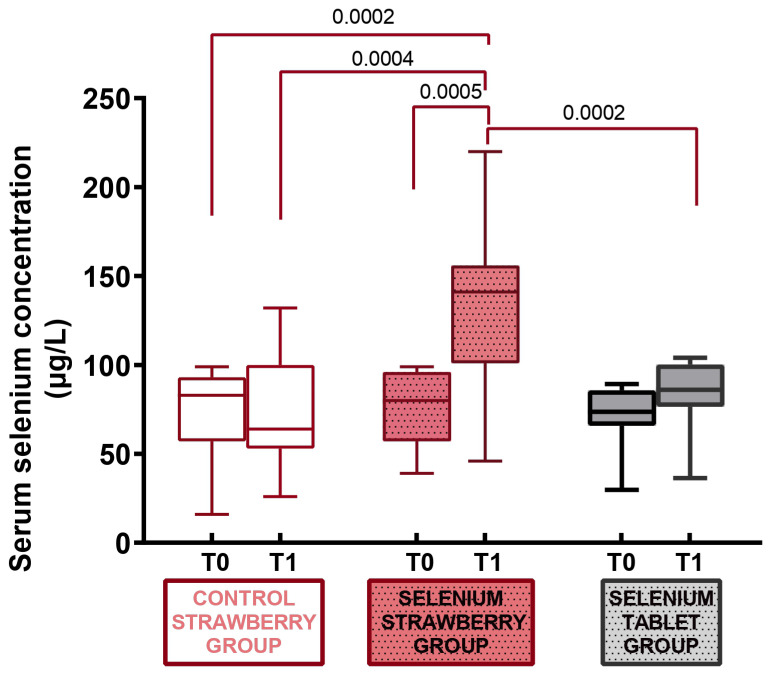
Concentration of serum selenium measured before (time 0) and at the end (time 1) of the intervention (30 days) in the control group, the selenium-biofortified strawberry group (100 g/day) and the selenium supplement group (tablet of 100 µg/day).

Serum selenium concentration was analysed following the 30-day intervention among the three groups (control, biofortified selenium and selenium supplement). In the selenium-biofortified strawberry group, a significant increase was observed in the serum selenium concentration by 73.6% compared to the control group, from 76 ± 20.3 µg/L to 132 ± 45.7 µg/L (*p* = 0.004, Cohen’s d = 1.12), while in the control group, the consumption of strawberries for 30 days did not cause any significantly change in the concentration of blood selenium (Figure 3).

### 3.4. Nutritional Intervention with Selenium-Biofortified Strawberries and Glucose Metabolism

The impact of daily selenium-biofortified strawberry consumption on glucose homeostasis was analysed. In the selenium-biofortified strawberry group, significant changes were observed in glucose metabolism markers. Specifically, a statistically significant reduction in fasting glucose levels was recorded, decreasing from 94 ± 5 to 84 ± 8 mg/dL (*p* = 0.0002, Cohen’s d = −1.53), corresponding to a 10.6% decrease. Fasting insulin showed a significant decrease from 9.6 ± 2.8 to 6.1 ± 2 mUI/L (*p* = 0.0242, Cohen’s d = −1.19), approximately a 57.3% reduction. Concurrently, HOMA-IR significantly decreased from 1.3 ± 0.3 to 0.8 ± 0.3 (*p* = 0.0178, Cohen’s d = −1.25), representing an approximate 38.5% reduction. Conversely, insulin sensitivity (HOMA %S) significantly increased from 88 ± 32 to 149 ± 61 (*p* = 0.002, Cohen’s d = 1.04), an increase of approximately 69.3% (Table 4). In the control group, consumption of 100 g of non-biofortified strawberries for four weeks did not result in any significant changes in serum concentrations of blood glucose, insulin, HOMA-IR, or insulin sensitivity (all *p* > 0.9). Similarly, no significant variations were observed for any of these parameters in the group that received selenium tablets (Table 4). The corresponding effect-size estimates (Cohen’s d) for all outcome measures and confidence intervals are provided in Supplementary Table S1. A two-way repeated-measures ANOVA (time × group) confirmed a statistically significant time × group interaction for all primary outcomes (fasting glucose, insulin, HOMA-IR, HOMA-%S, AST, ALT, GGT and serum selenium; all *p* < 0.05, partial η^2^ ≥ 0.20), indicating that the changes observed over the intervention period differed significantly among the three groups and were driven by the selenium-biofortified strawberry group (Supplementary Table S2).

### 3.5. Nutritional Intervention with Selenium-Biofortified Strawberries and Liver Function

The effect of the nutritional intervention with selenium-biofortified strawberries on liver function was then verified. Daily consumption for 30 days influenced endogenous levels of liver enzymes. Specifically, in the selenium-biofortified strawberry group, a significant reduction in AST was observed, decreasing from 22.5 ± 5.5 to 17 ± 2.7 U/L (*p* = 0.01, Cohen’s d = −0.82), representing an approximate 24.4% reduction. ALT also showed a significant decrease, from 18 ± 8 to 8 ± 2 U/L (*p* = 0.04, Cohen’s d = −1.31), indicating an approximate 55.6% reduction. Furthermore, GGT levels were significantly reduced from 15.6 ± 4.9 to 8.3 ± 2.3 U/L (*p* = 0.002, Cohen’s d = −1.35), an approximate 46.8% decrease, with all values remaining within physiological levels. The consumption of biofortified strawberries did not significantly affect albumin levels (*p* = 0.8, Cohen’s d = −0.35) (Table 5). In contrast, the daily consumption of control strawberries for four weeks did not determine any significant change in the serum concentrations of AST, ALT, GGT, and albumin (all *p* > 0.6). Similarly, no significant variations were observed for these parameters in the group that received selenium tablets (all *p* > 0.9) (Table 5). The corresponding effect-size estimates (Cohen’s d) for all outcome measures are provided in Supplementary Table S1.

The serum selenium concentration was analysed following the 30-day intervention among the three groups (control, biofortified selenium and selenium supplement). In the selenium-biofortified strawberry group, a significant increase was observed in the serum selenium concentration by 73.7% compared to the control group, from 76 ± 20.3 µg/L to 132 ± 45.7 µg/L (*p* = 0.004, Cohen’s d = 1.12), while in the control group, the consumption of strawberries for 30 days did not cause any significantly change in the concentration of blood selenium (Figure 3).

## 4. Discussion

This study analysed the impact of a 30-day nutritional intervention with selenium-biofortified strawberries in a cohort of healthy adults. Our findings suggest that daily consumption of biofortified strawberries is safe for the study population and may support maintenance of metabolic homeostasis.

For the study, we chose a homogenous cohort of participants. In order to avoid bias in lifestyle, including physical activity, the nutritional intervention was chosen according to a previous study [12]. The volunteers recorded the types and quantities of food and beverages consumed in a food diary, beginning one week before the start of the protocol and ending on the last day of the intervention. The selection of a biofortified vegetable matrix, specifically strawberries, offers distinct advantages. Strawberries are widely consumed fresh, are well accepted by participants, and are easy to integrate into daily diets, characteristics that significantly enhanced participant compliance and minimized study interruption. This approach aligns with sustainable agricultural practices and provides a palatable method for delivering essential micronutrients [16]. Firstly, the concentration of selenium in the crops was assessed to verify the efficacy of the biofortification process. The biofortification treatment was able to increase the selenium levels in the strawberries by 418.5% compared to untreated crops. Then, the effect of the consumption of biofortified products on the endogenous selenium serum levels of healthy volunteers was investigated. Attention should be given to selenium intake due its complex role in T2DM, showing both beneficial and harmful effects depending on the dose [17]. Endogenous selenium exhibits a U-shaped relationship with diabetes risk, where levels below 97.5 µg/L or above 132.5 µg/L are associated with increased susceptibility [17]. Since endogenous selenium follows a U-shaped curve, supplementation is indicated to be unnecessary when blood selenium levels reach approximately 125 μg/L [18,19]. The population of the study, consisting of healthy adults living in Sicily, exhibited baseline endogenous selenium levels of 73 µg/L. Daily supplementation of selenium (140 µg/day of selenium provided by biofortified strawberries) raised the value to 132 µg/L (+73.6%) and within physiological levels. This value is well below the tolerable upper intake level for selenium, which is 400 µg/day per day [20]. In this population, consuming selenium-biofortified strawberries modulated metabolic biomarkers by significantly lowering fasting glucose, insulin levels, and insulin resistance while enhancing insulin sensitivity. No such changes were observed in the control group. This indicates that increasing endogenous selenium levels through biofortified strawberry consumption may support glucose homeostasis. It is true that in our nutritional intervention, glucose levels decreased significantly within a safe range. Essentially, both baseline and post-intervention fasting glucose values remained within the physiological range (75 mg/dL to 100 mg/dL). However, several studies show that fasting glucose values in the low 80s are generally associated with lower future risk of insulin resistance and type 2 diabetes than values close to or higher than 90 [21,22,23]. These results should not be seen as a sign of curing an impaired glycaemic state but rather as an indication that selenium-enriched strawberries may preserve glucose-related metabolic parameters within a physiological range. With regard to the mechanism of action, a biological hypothesis provided by the literature says that the vegetable matrix may affect glycaemic and hepatic control at different levels of regulation [15,16,19,20]. In fact, this micronutrient influences insulin sensitivity via multiple mechanisms. Selenium is an essential requirement to produce the active antioxidant enzyme glutathione peroxidase (GPx), which protects cells from oxidative stress. When oxidative stress impairs insulin signalling, the cells become less responsive to insulin. Thus, the increase in the endogenous levels of selenium may allow the enzyme to work properly, reducing oxidative stress and promoting the maintenance of proper insulin signalling pathways and therefore glycaemic control. Previous studies in populations with conditions like polycystic ovary syndrome or obesity [24,25] did not find any improvement in glucose control following selenium administration, suggesting that micronutrients like selenium might exert their beneficial effects more effectively in the prevention of metabolic dysregulation rather than in managing established disease conditions [26,27,28,29].

However, we must point out that the novelty of the study lies in the successful delivery of selenium via a biofortified vegetal matrix in a healthy cohort. In fact, the selenium tablet group, despite receiving a daily selenium supplement (100 µg/day), did not exhibit similar beneficial effects on glucose homeostasis or hepatic function markers. In a study involving pregnant women with diabetes, the use of 200 µg/day of selenium supplementation for 6 weeks showed beneficial effects on glucose metabolism [30]. However, the explanation for this disparity lies in the dosage and times of treatment. Furthermore, the beneficial effects observed with biofortified strawberries may extend beyond the mere quantity of selenium. Selenium delivered within a complex food matrix, such as strawberries, likely benefits from synergistic interactions with other naturally occurring compounds (e.g., vitamins, fibre, and polyphenols) that can enhance its bioavailability, absorption kinetics, or overall metabolic impact.

Previous studies have demonstrated that selenium biofortification can significantly modify plant metabolism beyond selenium accumulation. In particular, metabolomic analyses in strawberries have shown an increase in flavonoids and polyphenols, as well as changes in fruit quality and antioxidant capacity [31]. Selenium biofortification has been reported to enhance antioxidant capacity and modulate the synthesis of secondary metabolites, including phenolic compounds and flavonoids, in various horticultural crops [32]. These compositional changes may enhance the nutraceutical properties of biofortified foods and contribute to their biological effects.

Selenium is part of the protein Selenoprotein P, which plays a role in insulin sensitivity by influencing glucose metabolism in the liver [33]. Therefore, the effects of selenium-biofortified strawberry consumption on key liver enzymes involved in glucose metabolism, specifically ALT, AST and GGT, were investigated [34,35]. The findings showed a beneficial impact on hepatic function. The consumption of selenium-biofortified strawberries induced a reduction by about 24% in AST levels, by about 55% in ALT levels and by about 47% in GGT. Notably, GGT is used as an indicator of liver health and is considered a stronger predictor of diabetic complications [36].

A limitation of the present study is that the selenium content provided by the biofortified strawberries (~140 μg/day) was higher than that supplied by the selenium tablets (100 μg/day), preventing a clear discrimination between dose-dependent and matrix-dependent effects. However, the inclusion of both a control strawberry group and a commercially available selenium supplement group was intended to control plant matrix effects and to provide a real-world comparison with conventional supplementation. Future studies using dose-matched interventions are warranted.

Regarding the lack of significant effects observed in the selenium tablet group, despite the benefits seen with biofortified strawberries, this finding suggests that the observed metabolic effects may not be explained by selenium dose alone. A plausible explanation lies in the different biochemical forms and the delivery context of selenium. In biofortified strawberries, selenium is predominantly present in organic forms (such as selenomethionine), which are known to have higher bioavailability and to be more efficiently incorporated into metabolic pathways compared to some inorganic or isolated supplemental forms [37,38].

Moreover, the food matrix likely plays a critical role. The complex matrix of strawberries may modulate selenium absorption, distribution, and retention, potentially enhancing its biological activity [39]. In addition, strawberries are naturally rich in polyphenols, vitamin C, and other bioactive compounds, which could act synergistically with selenium to influence redox balance, glucose metabolism, and hepatic function. These interactions are not replicated when selenium is administered as a single isolated compound in tablet form [40].

Therefore, the lack of effect in the selenium tablet group may reflect both differences in selenium speciation and the absence of matrix-related synergistic effects, rather than an absence of biological activity per se. The observed reduction in GGT by strawberry selenium supplementation further suggests improved liver glucose metabolism. In conclusion, the novelty of our study lies in the successful delivery of selenium via a biofortified vegetal matrix in a healthy cohort. 

## 5. Conclusions

This study underscores the potential of selenium-biofortified strawberries as a safe, palatable, and effective dietary strategy to positively modulate glucose and hepatic homeostasis, contributing to the prevention of metabolic dysregulation in healthy populations by using a sustainable agricultural process.

## Figures and Tables

**Table 1:** Dietary assessment of subjects in the three groups (control group, Se-biofortified strawberry group and selenium tablet group) during the 30-day treatment. All the values are indicated as means ± standard deviations (SDs). Statistical analyses performed via one-way ANOVA with Tukey’s post hoc test revealed no significant changes (*p* > 0.05, n.s.) among the three intervention arms.

Dietary Assessment	Control Group *n* = 12 Mean ± S.D.	Se Strawberry *n* = 12 Mean ± S.D.	Se Tablet *n* = 11 Mean ± S.D.	*p*-Value
Energy intake (kcal/day)	1950 ± 320	2051 ± 257	2020 ± 287	n.s.
Protein (g/day)	78 ± 15	82 ± 18	80 ± 19	n.s.
Carbohydrates (g/day)	230 ± 45	243 ± 64	245 ± 40	n.s.
Fats (g/day)	65 ± 18	63 ± 31	68 ± 15	n.s.
Cholesterol (mg/day)	240 ± 104.5	236± 88	255 ± 75	n.s.
Monounsaturated fatty acids (g/day)	25 ± 8	26 ± 14	27 ± 7	n.s.
Dietary fibre (g/day)	22 ± 7	24 ± 10	25 ± 6	n.s.
Water (Litres)	1.9 ± 0.2	2.1 ± 0.4	2.0 ± 0.1	n.s.

**Table 2:** Selection criteria of the study.

Criteria of Selection	Inclusion Criteria	Exclusion Criteria
Italian ethnicity, living in Palermo metropolitan area	Absence of gastrointestinal, cardiac and blood disfunction, absence of metabolic disorders, viral infection	Presence of chronic or metabolic disease
Not taking medications and supplements	Age range: 18–65 years	Use of supplements or medications
Clinically healthy	Body mass index: 18.5–27 kg/m^2^	Pregnancy, breastfeeding

**Table 3:** Characteristics of the participants. Values are expressed as mean and standard deviation (SD). A *p*-value > 0.05 indicates that there is no significant change.

Characteristics of the Study Groups	Adults n°12 (7 Females, 5 Males) Age Range 24–64 Years Old			Adults n°12 (7 Females, 5 Males) Age Range 25–65 Years Old			Adults n°11 (6 Females, 5 Males) Age Range 24–63 Years Old		
	Control Strawberry Group			Selenium Strawberry Group			Selenium Supplement Group		
	T0 Mean ± SD	T1 Mean ± SD	*p*-Value	T0 Mean ± SD	T1 Mean ± SD	*p*-Value	T0 Mean ± SD	T1 Mean ± SD	*p*-Value
Age (years)	40 ± 15		0.9	40 ± 17		0.9	42 ± 13		0.9
Height (cm)	167 ± 9		0.9	168 ± 7		0.9	173 ± 14		0.7
Weight (kg)	68.3 ± 11.4	68.3 ± 11.2	>0.9	69.3 ± 13	69.1 ± 13	>0.9	72 ± 10	72 ± 10	>0.9
BMI (kg/m^2^)	24.5 ± 3.4	24.5 ± 3.5	>0.9	25.5 ± 4.3	25.5 ± 4.3	>0.9	24 ± 3	24 ± 3	0.9
Fat mass (%)	30.4 ± 7.8	32.1 ± 6.9	0.98	30.5 ± 10	30.5 ± 10	>0.9	31.3 ± 3	31.3 ± 3	>0.9
Muscle mass (%)	30.7 ± 4.2	29.8 ± 3.8	0.99	31.0 ± 6.0	31.2 ± 6.0	>0.9	31.0 ± 5.9	31 ± 5.8	>0.9

**Table 4:** Markers of glucose homeostasis measured before (time 0) and at the end (time 1) of the nutritional intervention (30 days) in the control group, the selenium-biofortified strawberry group (100 g/day) and the selenium supplement group (tablet of 100 µg/day).

Glycaemic Profile	Adults n°12 (7 Females, 5 Males) Age Range 24–64 Years Old			Adults n°12 (7 Females, 5 Males) Age Range 25–65 Years Old			Adults n°11 (6 Females, 5 Males) Age Range 24–63 Years Old		
	Control Strawberry Group			Selenium Strawberry Group			Selenium Supplement Group		
	T0 Mean ± SD	T1 Mean ± SD	*p*-Value	T0 Mean ± SD	T1 Mean ± SD	*p*-Value	T0 Mean ± SD	T1 Mean ± SD	*p*-Value
Glycaemia (mg/dL)	91 ± 5	90 ± 7	0. 9	94 ± 5	84 ± 8	0.0002	90 ± 8	90 ± 6	>0.9
Insulin (mUI/L)	9.8 ± 4	9.6 ± 3	>0.9	9.6 ± 2.8	6.1 ± 2	0.0242	9.8 ± 3	9.6 ± 2	>0.9
HOMA–IR	1.2 ± 0.4	1.2 ± 0.4	>0.9	1.3 ± 0.3	0.8 ± 0.3	0.0178	1.2 ± 0.3	1.2 ± 0.2	>0.9
HOMA %β	106 ± 24	111.1 ± 24	0.9	100 ± 16	94 ± 20	0.9935	111 ± 28	113 ± 21	>0.9
HOMA %S	88 ± 28	89 ± 31	>0.9	88 ± 32	149 ± 61	0.002	88 ± 37	82 ± 17	>0.9

**Table 5:** Markers of hepatic function measured before (time 0) and at the end (time 1) of the nutritional intervention (30 days) in the control group, the selenium-biofortified strawberry group (100 g/day), and the selenium supplement group (tablet of 100 µg/day). AST: aspartate aminotransferase; ALT: alanine aminotransferase; GGT: gamma-glutamyl transferase.

Hepatic Function	Adults n°12 (7 Females, 5 Males) Age Range 24–64 Years Old			Adults n°12 (7 Females, 5 Males) Age Range 25–65 Years Old			Adults n°11 (6 Females, 5 Males) Age Range 24–63 Years Old		
	Control Strawberry Group			Selenium Strawberry Group			Selenium Supplement Group		
	T0 Mean ± SD	T1 Mean ± SD	*p*-Value	T0 Mean ± SD	T1 Mean ± SD	*p*-Value	T0 Mean ± SD	T1 Mean ± SD	*p*-Value
AST U/L	22.6 ± 4.2	23.2 ± 4.3	0.9	22.5 ± 5.5	17 ± 2.7	0.01	22.1 ± 3.9	22 ± 1.9	>0.9
ALT U/L	18 ± 9.0	19 ± 12	>0.9	18 ± 8	8 ± 2	0.04	20 ± 7	22 ± 9.6	0.9
GGT U/L	15.6 ± 4.3	15.6 ± 3.5	>0.9	15.6 ± 4.9	8.3 ± 2.3	0.002	14.3 ± 6.4	14.6 ± 6	>0.9
Albumin g/L	46 ± 3.2	44 ± 3.2	0.6	48 ± 3.6	46.3 ± 4	0.8	47.7 ± 1.6	47.5 ± 1.8	>0.9

## Data Availability

The original contributions presented in the study are included in the article/Supplementary Materials, further inquiries can be directed to the corresponding author.

## Supplementary Materials

The following supporting information can be downloaded at: https://www.mdpi.com/article/10.3390/nu18132078/s1, Table S1: Primary outcomes table: Mean difference (Δ), 95% confidence interval (CI), and effect size (dz, Cohen’s d for paired data); Table S2: Two-way repeated-measures ANOVA for the primary outcomes.
