# Supplementary material for: Selenium-Biofortified Strawberries Improve Glucose Homeostasis and Hepatic Function: A 30-Day Randomized Controlled Trial in Healthy Adults
Source: Nutrients. 2026 Jun 25;18(13):2078. doi: 10.3390/nu18132078 (PMC13362693; doi:10.3390/nu18132078)
Supplement: Supplementary file 1 [file nutrients-18-02078-s001.zip › nutrients-4372925-supplementary.pdf]

Table S1. Primary outcomes table: Mean difference (Δ), 95% confidence interval (CI), and effect size (dz, Cohen's d for paired data). The analysis compares pre-post (T1 − T0) intra-group changes. The groups are indicated along different lines.

**Outcome**	**Group**	**T0 (mean ± SD)**	**T1 (mean ± SD)**	**Mean Δ (95% CI)**	***p***	**d (dz)**
**Fasting glucose (mg/dL)**	Se-biofortified strawberries	94.1 ± 4.6	82.4 ± 6.9	−11.67 (−16.52, −6.81)	**<0.001**	**−1.53**
	Se tablets	90.3 ± 7.6	90.0 ± 6.1	−0.30 (−5.62, 5.02)	0.901	−0.04
	Control (strawberries)	90.9 ± 4.9	89.8 ± 7.0	−1.17 (−4.99, 2.65)	0.515	−0.19
**Insulin (µU/mL)**	Se-biofortified strawberries	9.6 ± 2.8	6.1 ± 2.4	−3.47 (−5.32, −1.61)	**0.002**	**−1.19**
	Se tablets	9.8 ± 2.8	9.6 ± 1.6	−0.25 (−2.44, 1.95)	0.805	−0.08
	Control (strawberries)	9.8 ± 3.7	9.6 ± 3.0	−0.15 (−1.72, 1.42)	0.838	−0.06
**HOMA-IR**	Se-biofortified strawberries	1.3 ± 0.4	0.8 ± 0.3	−0.47 (−0.71, −0.23)	**0.001**	**−1.25**
	Se tablets	1.3 ± 0.4	1.3 ± 0.2	−0.01 (−0.32, 0.30)	0.944	−0.02
	Control (strawberries)	1.3 ± 0.5	1.2 ± 0.4	−0.03 (−0.24, 0.18)	0.784	−0.08
**HOMA-%S (sensitivity)**	Se-biofortified strawberries	88.2 ± 32.4	148.6 ± 61.3	60.43 (23.54, 97.32)	**0.004**	**1.04**
	Se tablets	87.3 ± 37.0	82.0 ± 16.8	−5.27 (−34.41, 23.87)	0.692	−0.13
	Control (strawberries)	87.7 ± 28.4	88.8 ± 30.5	1.15 (−12.78, 15.08)	0.859	0.05
**AST (U/L)**	Se-biofortified strawberries	22.5 ± 5.6	17.1 ± 2.7	−5.42 (−9.61, −1.22)	**0.016**	**−0.82**
	Se tablets	22.1 ± 3.9	22.0 ± 1.9	−0.10 (−2.93, 2.73)	0.938	−0.03
	Control (strawberries)	22.7 ± 4.2	23.3 ± 4.3	0.58 (−3.18, 4.35)	0.740	0.10
**ALT (U/L)**	Se-biofortified strawberries	17.9 ± 7.7	8.2 ± 1.9	−9.75 (−14.49, −5.01)	**<0.001**	**−1.31**
	Se tablets	19.8 ± 7.2	21.6 ± 9.6	1.80 (−2.30, 5.90)	0.347	0.31
	Control (strawberries)	17.6 ± 9.8	18.5 ± 11.8	0.91 (−7.71, 9.53)	0.819	0.07
**GGT (U/L)**	Se-biofortified strawberries	15.7 ± 4.9	8.3 ± 2.3	−7.33 (−10.79, −3.88)	**<0.001**	**−1.35**
	Se tablets	14.3 ± 6.5	14.6 ± 5.9	0.30 (−5.06, 5.66)	0.902	0.04
	Control (strawberries)	15.7 ± 4.3	15.7 ± 3.5	0.00 (−1.53, 1.53)	1.000	0.00
**Serum selenium (µg/L)**	Se-biofortified strawberries	76.5 ± 20.4	131.8 ± 45.6	55.36 (22.21, 88.51)	**0.004**	**1.12**
	Se tablets	71.6 ± 17.5	83.2 ± 19.6	11.60 (1.34, 21.86)	0.031	0.81
	Control (strawberries)	72.5 ± 25.5	75.7 ± 32.3	3.27 (−29.41, 35.96)	0.828	0.07

Table S2. Two-way repeated-measures ANOVA for the primary outcomes. The time × group interaction (highlighted) is statistically significant for all primary outcomes, indicating that the pre–post change differed significantly between groups.

Outcome	Source of Variation	F	df	*p*	η^2p
**Fasting glucose (mg/dL)**	Time	14.61	1, 31	**<0.001**	0.320
	Group	0.59	2, 31	0.559	0.037
	**Time × Group**	**9.33**	2, 31	**<0.001**	**0.376**
**Insulin (μU/mL)**	Time	7.80	1, 31	**0.009**	0.201
	Group	2.29	2, 31	0.118	0.129
	**Time × Group**	**5.24**	2, 31	**0.011**	**0.253**
**HOMA-IR**	Time	7.58	1, 31	**0.010**	0.196
	Group	2.31	2, 31	0.116	0.130
	**Time × Group**	**5.55**	2, 31	**0.009**	**0.264**
**HOMA-%S**	Time	7.49	1, 31	**0.010**	0.195
	Group	4.22	2, 31	**0.024**	0.214
	**Time × Group**	**8.19**	2, 31	**0.001**	**0.346**
**AST (U/L)**	Time	3.15	1, 31	0.086	0.092
	Group	3.97	2, 31	**0.029**	0.204
	**Time × Group**	**3.91**	2, 31	**0.031**	**0.201**
**ALT (U/L)**	Time	2.82	1, 30	0.103	0.086
	Group	3.31	2, 30	0.050	0.181
	**Time × Group**	**5.54**	2, 30	**0.009**	**0.270**
**GGT (U/L)**	Time	7.36	1, 31	**0.011**	0.192
	Group	2.82	2, 31	0.075	0.154
	**Time × Group**	**7.51**	2, 31	**0.002**	**0.326**
**Serum selenium (μg/L)**	Time	10.52	1, 29	**0.003**	0.266
	Group	7.39	2, 29	**0.003**	0.338
	**Time × Group**	**4.97**	2, 29	**0.014**	**0.255**

η^2p = partial eta-squared; df = degrees of freedom (effect, error). Significant p-values (< 0.05) are shown in bold. The time × group interaction row is highlighted. Total analysed N = 32–34 depending on the outcome (paired observations).
